# Universal Verification Platform and Star Simulator for Fast Star Tracker Design

**DOI:** 10.3390/s21030907

**Published:** 2021-01-29

**Authors:** Victor Hugo Schulz, Gabriel Mariano Marcelino, Laio Oriel Seman, Jeferson Santos Barros, Sangkyun Kim, Mengu Cho, Gabriel Villarrubia González, Valderi Reis Quietinho Leithardt, Eduardo Augusto Bezerra

**Affiliations:** 1 Space Technology Research Laboratory (Space Lab), Universidade Federal de Santa Catarina (UFSC), Florianópolis 88040-900, Brazil; gabriel.marcelino@spacelab.ufsc.br (G.M.M.); eduardo.bezerra@ufsc.br (E.A.B.); 2 Graduate Program in Applied Computer Science, University of Vale do Itajaí (UNIVALI), Itajaí 88302-901, Brazil; laio@univali.br; 3 Department of Electrical Engineering, União Metropolitana de Educação e Cultura, Itabuna 42700-000, Brazil; jefersonengeletricista@gmail.com; 4 Laboratory of Spacecraft Environment Interaction Engineering (LaSEINE), Kyushu Institute of Technology, Fukuoka 804-8550, Japan; kim.sangkyun571@mail.kyutech.jp (S.K.); cho@ele.kyutech.ac.jp (M.C.); 5 Expert Systems and Applications Lab, Faculty of Science, University of Salamanca, Plaza de los Caídos s/n, 37008 Salamanca, Spain; gvg@usal.es; 6 VALORIZA, Research Center for Endogenous Resources Valorization, Instituto Politécnico de Portalegre, 7300-555 Portalegre, Portugal; valderi@ipportalegre.pt

**Keywords:** star tracker, verification, star simulator, field-programmable-gate-arrays, Universal Verification Methodology

## Abstract

Developing star trackers quickly is non-trivial. Achieving reproducible results and comparing different algorithms are also open problems. In this sense, this work proposes the use of synthetic star images (a simulated sky), allied with the standardized structure of the Universal Verification Methodology as the base of a design approach. The aim is to organize the project, speed up the development time by providing a standard verification methodology. Future rework is reduced through two methods: a verification platform that us shared under a free software licence; and the layout of Universal Verification Methodology enforces reusability of code through an object-oriented approach. We propose a black-box structure for the verification platform with standard interfaces, and provide examples showing how this approach can be applied to the development of a star tracker for small satellites, targeting a system-on-a-chip design. The same test benches were applied to both early conceptual software-only implementations, and later optimized software-hardware hybrid systems, in a hardware-in-the-loop configuration. This test bench reuse strategy was interesting also to show the regression test capability of the developed platform. Furthermore, the simulator was used to inject specific noise, in order to evaluate the system under some real-world conditions.

## 1. Introduction

The number of nanosatellites and picosatellites being launched has increased over the years. Associated with this growth, these smaller satellites have incrementally replaced functions previously only performed by bigger satellites by means of miniaturization of their components. The reduced physical volume also implies in smaller solar panels and batteries being employed, resulting in stricter energy constraints for the satellite subsystems. These constraints have brought a demand for technology development toward optimizing size, mass and energy consumption of CubeSat components [1,2].

As an example, which is the focus of this work, a subsystem might be responsible for attitude determination, finding the satellite’s orientation in space concerning a given reference system. Reliable attitude information is required, among other uses, for pointing the satellite’s solar panels towards the Sun and its antennas to the Earth. Several types of attitude sensors can be used alone or associated in order to obtain complimentary measurements and redundancy. Examples of commonly used ones are magnetometers, Sun sensors, horizon sensors and star trackers [3,4,5].

During the development of star tracker algorithms, it is necessary to evaluate how well the system behaves. Significant input needs to be generated, and the output response needs to be checked. While traditionally, the implementation of the algorithms was done in software, there are recent examples where hardware implementations targeting reprogrammable logic were explored [6,7], bringing advantages in terms of throughput.

Thus, the verification of star trackers has to consider the possibility of the system being composed of software, hardware, or a combination of these components. This ability is also important in this context of top-down design, when the engineer starts with a conceptual high-level software implementation that is progressively specialized and optimized for the target hardware and application.

Modern verification tools can be employed to address these emerging challenges. The Universal Verification Methodology (UVM), IEEE 1800.2 standard, applied to the SystemC family of libraries (https://accellera.org/activities/working-groups/systemc-verification/uvm-systemc-faq), offers a standardised structure for constructing an environment that supports the co-verification of software and hardware. The software part is usually written in the C++ language, and the hardware also described directly in C++, with the help of SystemC. More traditional hardware description languages (HDLs) such as VHDL (Very High Speed Integrated Circuit Hardware Description Language) and Verilog can also be used in co-verification. Once a test bench is created, it can be reused without changes during top-down design. Therefore, the algorithms under development can be directly compared, as they evolve. As opposed to traditional HDLs, multiple C++ libraries can be used to simplify the high-level development of the test benches or the system itself, saving time. UVM-SystemC is a recent development in the field of verification. It is currently a draft under public review, with its first public version being released in 2016 by Accellera.

In this context, this paper presents a verification platform for star tracker algorithms, following the structure of the Universal Verification Methodology standard. Making use of the UVM-SystemC environment paired with the OpenCV computer vision library, the platform can be used to test different algorithms for star trackers that perform centroid extraction, star identification [8] and attitude determination, separated or acting together. A universal design was achieved through a black box design and well-defined interfaces. Overall, the platform can be used as an aid to speed up development.

Considering the nature of the input images of the proposed system, a star simulator was developed to be able to synthesise the required stimulus—synthetic sky images with added controllable noise. A top-down design of mixed software and hardware components is explored as a practical application and proof of concept. Also, specific perturbations simulations can be performed. A prototype was submitted to satellite launch environment conditions, and the measured noise was used to configure the simulator.

In other words, this work has the main objective of proposing a design for miniaturised star trackers, with the focus on reducing the hardware requirements of the processing system. This reflects in smaller energy requirements through the appropriated sizing of the latter for a computational simpler task, which is of relevant importance considering its application in small satellites (i.e., through reducing the number of required software instructions, the microprocessors employed can be simpler; or can work with reduced frequency when idle). For this specific application, the development took in consideration the environmental conditions of LEO (Low Earth Orbit), with specific routines for tolerance to failure and noises being considered due to the existing adverse effects during operation.

In order to speed up the development, the system behavioural verification and the proposed improvements in software that aim for increased energy efficiency, as a secondary objective, a standardised verification system responsible for the synthesis of the inputs of the star tracker device was built. The environmental conditions of normal satellite operation, such as the introduction of specific noise patterns due to the exposition to solar radiation and variation in optical parameters of the lens/sensor set due to vibration and shock during launch were modelled and introduced into the simulation procedures performed by the verification system.

The remaining of the text is organized as follows. Section 3 describes how the star simulator and the interfaces relate to the verification platform’s general structure. Section 4 details the star simulator’s construction, including the proposed universal structures and configurable parameters. Section 5 presents the verification platform applications to the development of small star trackers and the obtained results. Section 6 discusses the conclusions and future works.

## 2. Related Works

There are already works where computer vision applications successfully employed UVM-SystemC as a design aid tool. In [9], a verification environment using SystemC and UVM was created for computational demanding video-based embedded systems. A system design starting with an executable specification in C++ and the computer vision library OpenCV ( https://opencv.org/) was verified. The system was progressively refined into lower levels of abstraction as an FPGA based smart camera. The final system works in a Zynq-7000 SoC, with the software part running in an ARM processor and the hardware part in the device’s programmable logic. Similarly, in [10] a UVM-SystemC environment was used to build a framework for the design and validation of face detection systems. Differently, no hybrid system is considered, but instead, a high-level model developed using OpenCV is used as the golden reference model, from which a complete hardware implementation of the system in SystemVerilog was compared.

Commonly, sky simulators allow the introduction of controllable noise to the system. For example, in [11,12] Gaussian noise was used to model random background noise. A more thorough model of the optical system of a star tracker, which includes the lens and image sensor, can be seen in [13]. The simulator considers the physical aspects of the optical system for emulating noise. Our verification platform employed a practical approach that uses simplified noise models (not much different from the former solutions). The mathematical functions for noise generation have their parameters tweaked in order to behave similarly to a physical optical system used as a reference. In the physical system, the noise was measured in conditions similar to what is expected during operation.

**Figure 1:**
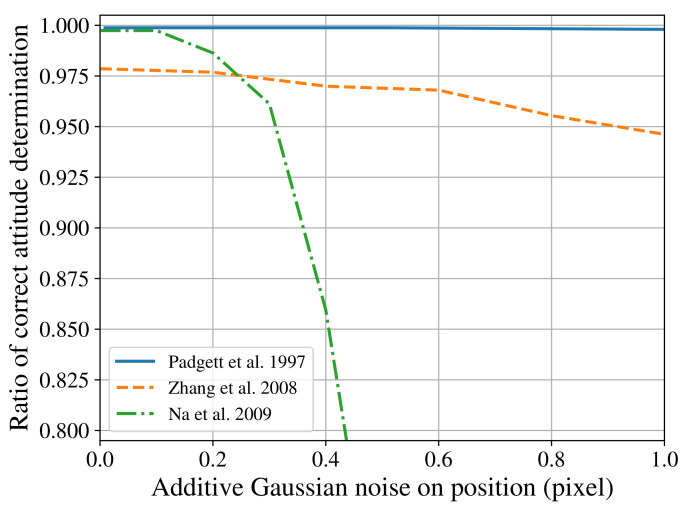
Effect of different test configurations on the behaviour of the Grid Algorithm. Padgett et al. [15] used $FOV=8^{\circ }\times 8^{\circ }$, res=512×512, $M_{max}=7.5$, g=40; Zhang et al. [16] used $FOV=12^{\circ }\times 12^{\circ }$, res=1024×1024, $M_{max}=6.0$, g=n/a; Na et al. [17] used $FOV=8^{\circ }\times 8^{\circ }$, res=n/a, $M_{max}=6.5$ and g=40.

In the usual way star tracker algorithms are evaluated, the images produced by a star simulator are employed as the input of one or more reference algorithms. The same input is then applied to the novel algorithm, and the results compared with the intent of showcasing improvements. Kolomenkin et al. [14], when discussing this matter, stated that this is not a trivial task, due to no agreed standard: “Many authors have referred to different aspects of star tracker performance such as speed, accuracy, memory requirements, and stability. But each of them used a different configuration”. The consequence of their observation can be seen in Figure 1. It shows three different authors’ [15,16,17] data on how the Grid algorithm [18] behaves with the presence of positional noise, using different test configurations for field of view (FOV), resolution (res), maximum visual magnitude ($M_{max}$), and grid size *g*. Different star simulators were also used, and the implementation of Grid likely differs between researches [19,20].

Instead of suggesting a given set of configurations that should be followed by all researchers when evaluating star trackers, this work provides a universal verification platform and star simulator. It has the flexibility of easily working with any desired conditions. Thus, the verification platform was built with configurable optical parameters such as sensor resolution, field of view and maximum visible star magnitude. Noise levels are also configurable. The aim is to easily test algorithms in similar conditions of those used in multiple, different previous researches. Effectively, this reduces the need to implement reference algorithms again.

Also, in contrast to other solutions, our simulator is implemented with the specific features for testing star tracker algorithms in mind, and, at the same time, built from ground up with a focus in reusability. Our solution is unique in the sense that it becomes the sequence generator component of a UVM structure, which standardizes the way the test cases are built. The standard package simplifies the interfaces between the components under the test and the UVM test bench in a black box construction, so that it becomes simple to interface star tracker algorithms to the developed verification environment.

Table 1 shows a summary of the presented star simulators based on information published, as none of them are available online, and neither are published as free software. There are optical simulators for hardware-in-the-loop test of complete star trackers, that while are out of the focus of this work, are also available in the literature [21,22,23,24].

## 3. Universal Verification Platform

**Figure 2:**
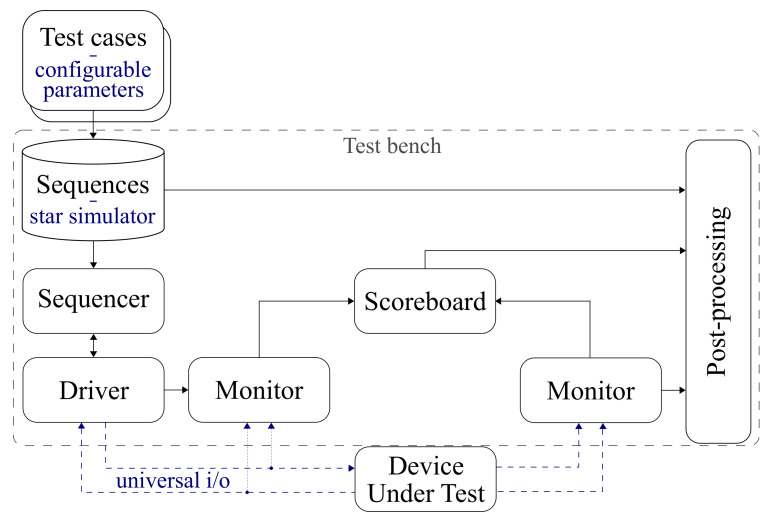
Structure of the verification platform. Following Universal Verification Methodology (UVM) nomenclature, the composing blocks are linked to their specialised functionalities for the particular case of verifying star trackers.

The proposed verification platform follow the structure and terminology of UVM-SystemC [29]. Figure 2 links UVM components with their specialised functionality needed for the verification of star trackers.

The main function of the test bench in UVM consists of the interaction with the Device Under Test (DUT), through injection and data collection on the created interfaces, according to the desired test cases. The test bench components are hierarchically separated, and they assume different tasks.

The creation of test data is performed at a high-level of abstraction through the aid of C++ features, mathematical functions, and libraries. This feature of UVM-SystemC allowed the creation of a complete star simulator (Section 4) to generate input for either the entire DUT or its separate components. In our implementation, the star simulator corresponds to the sequences component, and the configurable parameters (Section 4.3) define the test cases.

The data generated by the simulator are then delivered to the driver component. It will receive the items, translating or adapting them for the DUT interface. Furthermore, the input and output monitors behave like testing probes, accessing the DUT interface and capturing relevant information.

The scoreboard component is then responsible for comparing the outputs of the system with the reference, effectively producing scores, which are post-processed as required. The post-processing consists, for example, of analyzing the scores produced by UVM for generating tables and plotting graphics. In the case of the current implementation, the scoreboard data is written to a file. The data is then automatically processed by an auxiliary program written in Python. The plots displayed later on Section 5 are examples of the post-processing.

**Figure 3:**
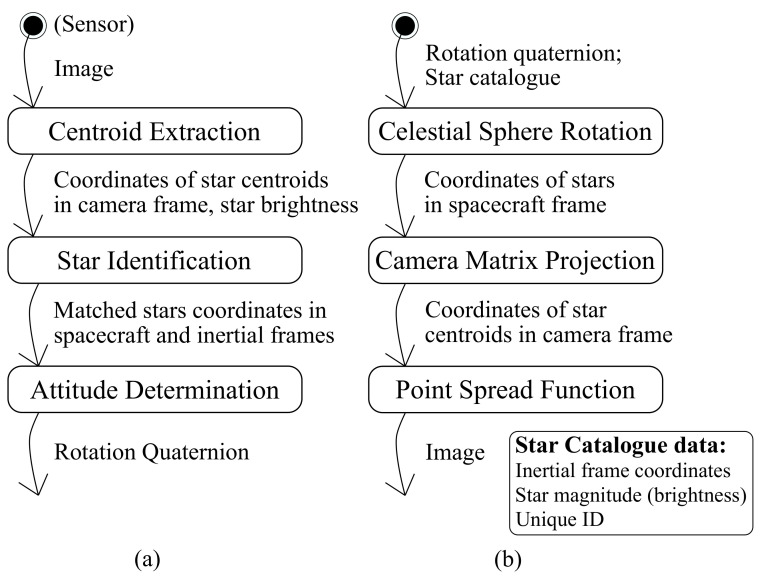
(**a**) Star tracker software input and output; and (**b**) star simulator stages. The boxes indicate the software components, and the arrows indicate the data flow between them.

The verification platform is very flexible in generating data and testing different DUTs. Such flexibility was possible due to the creation of a universal data packet. The packet encapsulates the universal i/o structures, generated by the sequences block. The packets are then transmitted to the interfaces of the DUT. They contain input information that can be used by the individual components (Figure 3), whether working together as a whole or separately.

The transportation of these packets to the DUT was also standardized with the TLM 2.0 [30] communication standard. This abstract communication pattern transports the data without the need for further detail on how the data would be transported in the real system.

Two distinct DUT wrappers were developed by us. The first interfaces the TLM packets from C++ structures to VHDL, and the second transport the structures through TCP/IP interfaces. They were used to demonstrate the verification of VHDL components for FPGA designs and hardware-in-the-loop designs, respectively.

As all the components are implemented with the object-oriented paradigm, they can easily be reused between tests to the extent to which it makes sense. This is made possible by the inheritance and polymorphism features of the C++ language. The UVM structure enforces the test bench’s organization so that it becomes progressively more straightforward to implement new test cases, as most structures can be reused.

The base element of the UVM test bench is the Transaction Level Modelling (TLM). These transactions are communications between functional blocks and encapsulate data that can be information packages, for example the address and data in a serial communication protocol, without exactly containing the information of how this data would be delivered to the DUT. Within the analogue domain, packages can contain data for configuring a sinusoidal power source, sawtooth, and so forth. This allows the generation of input data with the freedom of working in higher levels of abstraction, without having to worry with the details of the signal level [31].

Thus, the UVM is well structured to serve as the fundamental base for implementing the proposed testing platform. It enables the testing platform to follow a well-documented structure, the easy implementation of the proposed tests and can be expanded and extended easily to implement future tests by the community. By pairing the UVM standard with SystemC, it is also possible to apply the same testing platform to different DUTs, and support both software, hardware and mixed environments in co-verification.

## 4. Star Simulator

The general mathematical model used during the star simulator development simulator is similar to those presented in [11,12]. The main difference is in the way the rotations are performed: we used quaternions instead of Euler’s rotation matrices. The use of quaternions simplifies the equations and avoids gimbal lock limitations.

The main building blocks of a star tracker and a star simulator can be seen in Figure 3. Note that the star tracker’s basic flow (a) has a dual structure compared to the star simulator structure (b), working in reverse order. The exception is that some of the data is provided directly by the star catalogue. The starting point of a simulator is the star catalogue. The extraction of relevant data from it is described next.

The algorithm blocks shown in Figure 3 will be using the same data structure as both their inputs and outputs, making the reconfiguration of test benches easier. The magnitude field can also be used for returning brightness data as long as the sign is inverted, as magnitude goes down while brightness goes up and vice versa. This ensures that posterior sort procedures on the algorithms work similarly.

Notice that this work does not take into account the digital image acquisition procedure (carried out by focal plane electronics, mainly detector and ADC). Thus, the issues related to the electronics, such as noise and temperature effects are not considered.

### 4.1. Star Catalogue

Star catalogs contain astrometric and photometric data, usually collected by observations of specialized satellites. In the context of star simulators and star identification algorithms, such data is used as the fundamental data for constructing the internal database of stars. In this work, the Hipparcos (http://cdsarc.u-strasbg.fr/viz-bin/Cat?I/239) star catalogue [32] was selected for use, due to its highly accurate data for brighter stars. Current CCD and CMOS sensors are able to detect stars around magnitude 6.0 and lower (counter-intuitively, lower values of star magnitude actually mean brighter stars) [33], making the selected catalogue appropriate. The Hipparcos-2 (http://cdsarc.u-strasbg.fr/viz-bin/Cat?I/311) catalogue [34] was a later improvement on the accuracy of original Hipparcos data, achieved by a new reduction of astrometric data. The updated data was considered in this work, complemented with information from the original Hipparcos catalogue when such data was not available on the updated version.

The entries of interest in Hipparcos and Hipparcos-2 catalogues are shown in Table 2. On the Table, the most important fields are RArad, DErad and Vmag. They correspond, respectively, to the right ascension coordinate, the declination coordinate, and the stars’ visual magnitude (brightness). The coordinates are represented in the International Celestial Reference System (ICRS), in the epoch 1991.25, defining the catalog’s inertial reference system. The Hipparcos identifiers are useful for pairing entries from the two catalogues. Proper motion information can be used to correct for the perceived movement of stars through time in the celestial sphere.

The entries of the catalogue which are above the threshold of magnitude considered, and thus deemed to be undetectable by the sensor, were eliminated. This greatly reduced the dimensions of the database constructed from the data. The star coordinates were updated to simulated space mission epoch through proper motion correction, then transformed from their angular representation (right ascension α and declination δ) into a unit vector (inertial) in Cartesian coordinates. The process is detailed in sequence.

Proper motion is defined as the time derivative of the positional coordinates of right ascension (α) and declination (δ), as:

(1a) $$\begin{array}{cc} \; & \mu_{\alpha }=\frac{d\alpha }{dt} \end{array}$$

(1b) $$\begin{array}{cc} \; & \mu_{\delta }=\frac{d\delta }{dt} \end{array}$$

The Hipparcos entries are pmRA ($\mu_{\alpha }*$) and pmDE ($\mu_{\delta }$). The asterisk in the proper motion of right ascension denotes that it is converted to great circle measurements for being directly comparable to $\mu_{\delta }$. It is necessary to undo this conversion before applying the corrections [32], by determining $\mu_{\alpha }$:

(2) $$\mu_{\alpha }*=\mu_{\alpha }\times \cos\left(\delta \right)$$

Before making the correction, the units for the proper motion variables must be converted from milliseconds of arc/year to radians/year, as:

(3a) $$\begin{array}{cc} \; & \mu_{\alpha }\left(rad/year\right)=\frac{\mu_{\alpha }\left(mas/year\right)\times \pi }{3600\times 1000\times 180} \end{array}$$

(3b) $$\begin{array}{cc} \; & \mu_{\delta }\left(rad/year\right)=\frac{\mu_{\delta }\left(mas/year\right)\times \pi }{3600\times 1000\times 180}. \end{array}$$

Then it is possible to correct the proper motion by using its definition Equation (1a), which is represented in Equation (4a). Here the time *t* unit is years.

(4a) $$\begin{array}{cc} \; & \alpha =\alpha +\mu_{\alpha }\times t \end{array}$$

(4b) $$\begin{array}{cc} \; & \delta =\delta +\mu_{\delta }\times t \end{array}$$

The new α and δ coordinates can then be transformed into unit vectors using the following equation:

(5) $$uv=\left[\begin{array}{c} \cos(\alpha )\times \cos(\delta )\\ \sin(\alpha )\times \cos(\delta )\\ \sin(\delta ) \end{array}\right]$$

### 4.2. Generating a Synthetic Star Image

After all the steps mentioned in Section 4.1 are applied to the catalogue, the result will be a celestial sphere composed of unit vectors. All stars above the magnitude threshold being considered will have a respective unit vector on the sphere [35].

The desired celestial sphere attitude is expressed in the form of a rotation quaternion, which can be known or randomly generated. Using quaternion multiplication, the original attitude of the reference system can be rotated into the desired attitude. Following the notation on [35], Equation (6) shows the form of the rotation quaternion q, and how it is constructed from a unit vector u, representing the desired axis of rotation, and the desired angle of rotation θ.

(6) $$q=\left[\begin{array}{c} q_{1:3}\\ q_{4} \end{array}\right]=\left[\begin{array}{c} \sin\left(\theta /2\right)\times u_{1:3}\\ \cos(\theta /2) \end{array}\right]$$

A 3D unit vector v (of a star) can be expressed in quaternion form according to Equation (7). The rotation itself is performed using Equation (8).

(7) $$p=\left[\begin{array}{c} v_{1:3}\\ 0 \end{array}\right]$$

(8) $$p^{\prime }=q⊗p⊗q^{*}$$

The superscript * denotes the conjugate of the quaternion, which is defined as:

(9) $$q^{*}={\left[\begin{array}{c} q_{1:3}\\ q_{4} \end{array}\right]}^{*}\equiv \left[\begin{array}{c} -q_{1:3}\\ q_{4} \end{array}\right]$$

The cross-product operator is given by:

(10) $$\bar{q}⊗q=\left[\begin{array}{c} q_{4}{\bar{q}}_{1:3}+{\bar{q}}_{4}q_{1:3}-{\bar{q}}_{1:3}\times q_{1:3}\\ {\bar{q}}_{4}q_{4}-{\bar{q}}_{1:3}\cdot q_{1:3} \end{array}\right]$$

After performing the same 3D rotation on all stars, a projection of the unit vectors ${\left[XYZ\right]}^{\prime }$ is made using the pinhole camera model Equation (11) into the virtual sensor image plane. Stars that do not have a projection lying in this plane are eliminated from the current simulation. In Equation (11), the left vector is represented using a homography coordinate system, thus it should be normalised by *w*. The resulting *x* and *y* coordinates represent the projection. In Equation (11), $\left(c_{x},c_{y}\right)$ are the centre of the sensor plane, in pixels, and $\left(f_{x},f_{y}\right)$ correspond to the focal length of the lens, also in pixels [35].

(11) $$\left[\begin{array}{c} x\\ y\\ w \end{array}\right]=\left[\begin{array}{ccc} f_{x} & 0 & c_{x}\\ 0 & f_{y} & c_{y}\\ 0 & 0 & 1 \end{array}\right]\times \left[\begin{array}{c} X\\ Y\\ Z \end{array}\right]$$

Finally, a Point Spread Function (PSF) is used to simulate the spreading of the light upon multiple pixels, and the virtual image is formed. Equation (12) shows the PSF used in [11]. The simulator designed in this work uses an adapted version of this equation.

(12) $$I\left(m,n\right)=\int_{m\;dx}^{(m+1)\;dx}\int_{n\;dy}^{(n+1)\;dy}\sum_{i=i}^{N}\frac{C}{2.512^{M_{i}}}\times \exp\left(-\frac{{\left(x-\bar{X_{i}}\right)}^{2}+{\left(y-{\bar{Y}}_{i}\right)}^{2}}{2\sigma^{2}}\right)dy\;dx+B$$

where:

- I(m,n) = Pixel value function;
- (m,n) = Pixel coordinates (discrete);
- $M_{i}$ = Magnitude of *i*-th star;
- (x,y) = 2D sensor frame coordinates (continuous);
- $\left({\bar{X}}_{i},{\bar{Y}}_{i}\right)$ = Positional mean;
- σ = Positional standard deviation.
- *B* = Constant.
- *C* = Constant;

The constant parameter’s value can take the appropriate values as required based on the design specifications of the optical system and image device [11]. Also, notice that the PSF used here does not take in account the effect of aberrations, which should be considered for a more precise outcome [36,37].

While the Gaussian function for a single star can be evaluated at all (m,n) pixels, to increase the simulator’s performance, a configurable window is used to limit the neighboring pixels considered for each star [11].

Equation (12) can be expressed in terms of the error function erf(x) as:

(13) $$\begin{array}{c} I\left(m,n\right)=\frac{1}{2}D\pi \sigma^{2}\times \left(erf\left(\frac{n-{\bar{X}}_{i}}{\sqrt{2}\sigma }\right)-erf\left(\frac{1+n-{\bar{X}}_{i}}{\sqrt{2}\sigma }\right)\right)\times \left(erf\left(\frac{m-{\bar{Y}}_{i}}{\sqrt{2}\sigma }\right)-erf\left(\frac{1+m-{\bar{Y}}_{i}}{\sqrt{2}\sigma }\right)\right) \end{array}$$

**Figure 4:**
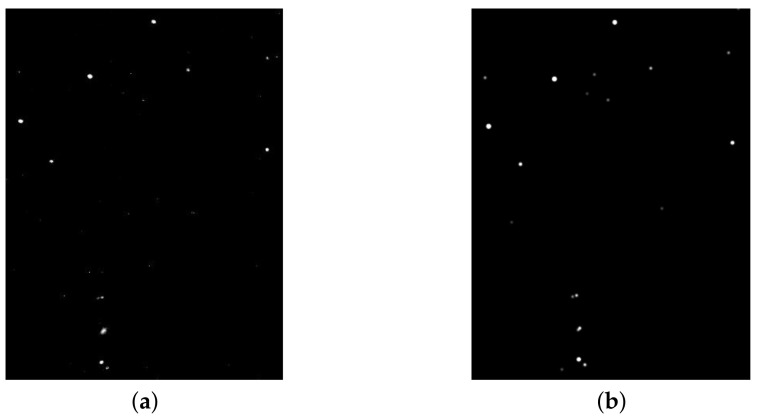
Comparison of (**a**) a real star image, from the ASTERIA CubeSat, JPL/NASA; with (**b**) a synthetic image generated by our star simulator. The images show part of the Orion constellation.

The mathematical model previously described can visually synthesize star images similar to images captured by real hardware. A visual comparison can be seen in Figure 4, where there is a comparison to an image taken from the ASTERIA CubeSat (https://www.nasa.gov/feature/jpl/astrophysics-cubesat-demonstrates-big-potential-in-a-small-package). Notice that although the star simulator manages to achieve results close to the real images, small differences can still be noticed in the image due to the simulated nature of the process.

Notice that, instead of projecting the observed stars on the focal length, one could calculate the sensor orientation in the star catalog reference system, select the catalog start within the sensor FOV, and then project only the viewed stars onto the sensor focal plane.

### 4.3. Configurable Parameters

To make the verification platform configurable, to allow simulations to be made in similar testing conditions of previous research, it was built so that the optical parameters of the virtual camera can be easily changed. As a way of presenting an example of application of the proposed solution, we will consider three parameters to define a given virtual camera. They are the field of view angle (FOV), the sensor’s resolution (res) and the sensibility of the sensor, represented by the maximum visual magnitude of stars ($M_{max}$) that can be detected. As a convention, we use the vertical axis for calculations (*y*) [38]. Notice that this approach includes limitations and does not generalize to modern star trackers’ operating conditions.

In the simulator, stars with higher magnitude than $M_{max}$ are excluded from the processing. To generate a virtual camera, a camera matrix with the form shown in Equation (11) is created. The centre values ($c_{x}$, $c_{y}$) correspond to half of the resolution components ($res_{x}/2$, $res_{y}/2$). Equation (14) defines how the focus distance, in pixels, can be calculated from the desired field of view angle ($FOV_{y}$) and the vertical resolution ($res_{y}$) [38].

(14) $$f=\frac{res_{y}}{2\tan(FOV_{y}/2)}$$

Noise can also be added and controlled in simulations with independent configurable parameters. The types of noise relevant to test each component of star tracker algorithms are discussed further in Section 4.4.

### 4.4. Noise Injection

Noise can manifest in star trackers in the form of smaller location accuracy of stars in the sensor plane, differences in perceived brightness (perceived stellar magnitude) or the presence of false stars. The reason for this tolerance is that working with binary patterns leaves some room for changes in the location of the stars while still generating the same pattern, and false stars only change a single bit, leaving the pattern still very close to the original. It is important to notice that today’s algorithms from the first class have shown to perform very well in the presence of false stars [14].

For a specific test case, the injection of controlled noise should be done according to the input being expected by the algorithm. Thus, the most adequate point where noise should be added depends on what algorithm is being tested [39,40]. When two adjacent algorithms are being tested (see Figure 3a), for example centroid extraction and star identification, or star identification and attitude determination, the former will define the appropriated input noise, while the latter will define the scoring procedures. From the layout shown in Figure 3b, the outputs of each stage are kept in the universal structures. The ones that correspond to the DUT’s input can have controlled noise added to them when required.

The quality of the image acquired by the sensor is affected, in a broad perspective, by changes in the bi-dimensional location of the star projections, or changes on the brightness of stars (especially ones close to the threshold of detection of the image sensor). The amount of background noise in the image can reduce the signal to noise ratio (SNR) and bring problems such as false stars being detected by the centroid extraction algorithm. Radial and tangential distortions of the lenses, along with chromatic aberration, can also make stars appear in shifted positions on the image plane. While some of these noises are systematic and could be minimized, for example, with a camera calibration in the laboratory, other noises can be random. Under real operating conditions the optical system could be subject to vibration or thermal variations, which would affect the lens focal length value and change the distortion pattern. Also, radiation total-dose and single-events can change the image, respectively, by raising its background noise (dark current) and by causing the emergence of hot pixels (which could mistakenly be detected as stars). Devices Under Test (DUTs) that expect an image as input will therefore need to consider these types of noises to be introduced in the image.

In the case of star identification being evaluated in a DUT, separated from the centroid extraction step, the system’s input becomes a list of star centroids with apparent brightness information. This corresponds to the output of the (now absent) centroid extraction step. Now, the effects of the previously mentioned noises can be considered directly: absence of stars that should be detectable; presence of false stars, or even stars that are above the expected detection threshold of magnitude; errors in estimation of apparent brightness; and positional errors in the star centroid estimation. This ultimately affects the number of total identified stars and how many of those were ultimately correctly identified.

When the DUT is composed of only the static attitude determination step, its input becomes a list of stars uniquely identified. Each entry of this list is associated to the star’s inertial and spacecraft centred coordinates. In this step, errors can happen due to a change in the position of the unit vectors in camera frame. They can also be caused by misidentifications when the inertial coordinates of unrelated stars could be associated with actual stars’ spacecraft coordinates.

### 4.5. Tests

A literature review of some of the existing tests performed by different authors was made, with the intuit of serving as candidates for composing the battery of tests of the platform. A summary is shown next:

- Single star centroid estimation:
  - Error vs. noise standard deviation [14].
- Star identification rate/successful attitude determination rate:
  - Percentage of correctly identified stars histogram [14];
  - Percentage of none/correct/ambiguous/wrong stars vs. position error [41];
  - Percentage of none/correct/ambiguous/wrong stars vs. brightness error [41];
  - With added false stars [14];
  - With 1 or 2 added false stars with brightness error [7];
  - With introduced position errors [7,15,16,17,18];
  - With introduced brightnes noise [7,15,16,17];
  - With introduced focal length deviation [15];
  - Percentage of correctly identified stars vs no. of stars in the FOV [16].
- Bore-sight error, roll error:
  - Error vs. number of tests histogram [14];
  - Error vs. correctly identified stars [14].
- System properties:
  - Runtime [7,14,15,18];
  - Runtime vs. no. of stars in the FOV [16];
  - Memory requirements [7,14,15,18].

The (*i*) single star centroid estimation [14] is a test that evaluates the performance of the centroiding component of a star tracker. The star identification step is evaluated either by the (*ii*) rate of correct identification of stars or the successful attitude determination [14], incorporating the attitude determination subsystem in the test. Both tests can be perturbed by the same kinds of noise, which can impact the algorithms in different ways. For example, there are algorithms which work with uncalibrated cameras [42], which are expected to have good performance with focal length deviation. (*iii*) Bore-sight errors [14] are a difference in angle between the determined attitude and the correct attitude, while the roll error is the rotation difference between them. These two tests can be applied to all parts of a star tracker. Finally, there are tests which display system properties, (*iv*) measuring the runtime of the algorithms in a given hardware and the memory consumption of the database [7,14,15,18]. The memory use can be theoretically calculated by analysing the data structures involved, but in practice both tests will present different results under different hardware and operating system environment conditions, so it is difficult to present an universal comparison.

The tests can have different results with different parameters, such as the field of view (which depends on the focal length) and the sensibility of the sensor. Both will affect the number of stars visible in similar orientation conditions, which can impact algorithms’ identification rates in different ways. Therefore, both wide and restrict field of view configurations should be considered, as done in the tests in [14].

## 5. Case Study

This section presents practical examples that demonstrate how the verification platform can be used as an aid in design. From the beginning, having the sky simulator, the skeleton of the verification platform, and the inputs and outputs well defined relieves the engineer’s initial work, allowing him/her to focus on the design of the star tracker only saving time. The benefits of the verification platform are not limited to the initial set-up though. The following three examples are going to be explored:

- Reproducing Existing Test Conditions: demonstrates the verification platform’s ability to work in different test configurations.
- Computational Hot Spot Optimisations: shows how the platform can be applied to effectively speed-up the design of star tracker through optimizations focused on the most demanding parts of the algorithms. The example focuses on reducing the runtime of the algorithms to reduce the energy requirements ultimately.
- Launch Environment Tests and Focal Length Noise: explores how the measurement of noise levels in real-world settings, measured in launch environmental tests, can be used to calibrate the noise levels of the star simulator. A test bench is constructed, which can then be used to evaluate star tracker algorithms in similar conditions through software simulations.

### 5.1. Reproducing Existing Test Conditions

**Figure 5:**
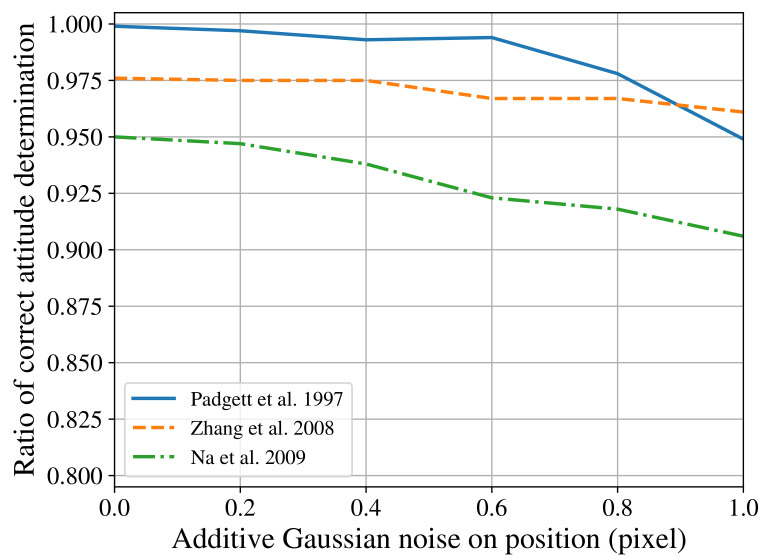
Behaviour of grid algorithm in different test configurations, which were reproduced from Padgett et al. [15]: FOV= 8 × 8 degrees, resolution = 512 × 512, Mv = 7.5, *g* = 40; Zhang et al. [16]: FOV = 12 × 12 degrees, resolution = 1024 × 1024, Mv = 6.0, *g* = 40; Na et al. [17]: FOV = 8 × 8 degrees, resolution = 512 × 512, Mv = 6.5 and *g* = 40.

To demonstrate the verification platform’s flexibility for working in different conditions, we submitted our implementation of the Grid algorithm to similar test conditions of previous research. The original results from these researches were shown previously in Figure 1. The reproduced results obtained with our platform can be seen in Figure 5. As some test condition parameters could not be found in the original articles, and due to differences in our implementation of the Grid algorithm and star simulator, some differences in the results obtained can be observed.

The main application of this capability is that, if working with the limitations is possible, significant time can be saved by avoiding the implementation of reference algorithms for comparisons. Instead, it becomes possible to use data from other researches directly. As the proposed platform is shared as free software, most differences in test conditions can be eliminated entirely in the future with the adoption of the same tools.

### 5.2. Computational Hot Spot Optimizations

This section shows how the verification platform can be applied to speed-up design. In short, the strategy employed is to implement a high-level software design of the system and identify regions of the program where most of the time is spent. These regions are called hot spots. When moving to lower-level implementations, the engineer only concentrates his/her attention to optimize the hot spots. This is done by redesigning the software with optimizations or specialized hardware acceleration (e.g., FPGA). This prevents excessive work on optimizing components that are not critical.

The steps followed for optimization were:

1. High-level implementation of a reference design;
2. A test bench is created to measure the runtime of software components;
3. Identification of hot spots with proportionally high runtime in the reference design;
4. A strategy is created for optimizing the hot spots;
5. Implementation of optimizations (software or hardware);
6. The same test bench for measuring runtime is applied to the optimized system, determining the effective changes.

Different test benches can also be used to ensure that the algorithm is still performing as expected for detection rates. The practical application of these steps is shown next.

#### 5.2.1. Runtime Analysis

The test bench created for the runtime tests considered a vision system with a resolution of 800×600, pixel size of 2.8µm, and two vertical field of view (FOV) configurations: 8 and 15 degrees. FOVs configurations used for evaluating star trackers vary between authors. The values considered were selected to simultaneously try to represent popular configurations and allow the observation of differences in the algorithms’ behavior when operating on narrower and wider angles. The pixel size and resolution are based on a real COTS sensor, the MT9D111, working at half its maximum resolution, aiming to simulate its operation. The lower resolution was chosen to facilitate debugging of hardware implementations of algorithms, due to limitations in integrated memory. The verification platform does not restrict these parameters; thus, the simulation conditions can be easily changed, and the simulations redone as required.

The algorithms used on the DUT were—the Region Growing [28] (for centroid extraction); the Grid Algorithm [18] (for star identification, with grid size g=24) and Quest [43] (for static attitude determination).The verification platform and star simulator ran in a personal computer, and a ZedBoard development kit with a dual-core ARM Cortex A9MP (ARM v71) Zynq-7000 SoC @ 667 MHz was running the algorithms in a single thread and behaving as the DUT. The hardware-in-the-loop was implemented using a TCP/IP communication channel between the two systems. The sequence considered was a thousand random attitude configurations. For each attitude, the corresponding sensor image was synthesized by the simulator. This test case was repeated 11 times, with the first time discarded, and the runtime of each sky configuration was measured in the kit using the Chrono time library included in the C++11 standard. The repetitions were performed in order to reduce the impact of random measurement noise on samples. The operating system used was GNU/Linux, with a non-real-time kernel. Table 3 shows the average and standard deviation for the sky configurations considered.

A high standard deviation was displayed in the runtime measurements of the star identification step. This is related to the variable number of stars that can be present in a random sky image. For the Grid Algorithm, that is, star identification, configurations that contain more stars will result in a longer processing time, as more catalogue lookups need to be performed. The algorithm’s rate of growth is discussed in more detail in Section 5.2.3.

Differently, only a small difference in runtime could be observed for the different fields of view for the centroid step. This is an indication that the threshold operation used for segmentation of the stars, which considers all the pixels, predominates over the calculation of the centroids itself. Thus, the algorithm employed is more sensible to the image’s resolution than to the number of stars present in it. The standard deviation observed also supports this interpretation: even though the number of stars was changing between images, the runtime remained almost constant.

Through the analysis of the mean and the standard deviation of the runtime values, and combining with the knowledge of the structure of the algorithms used, we located two hot spots of the system: the threshold operation of the centroid step; and the catalogue lookup operation of the star identification step.

#### 5.2.2. Improving the Centroid Extraction Step Performance

As can be seen in the results listed in Table 3, when working with a narrower FOV, the centroid extraction becomes the step with the highest consumption of resources. Thus, it is one possible target for optimisation when aiming for performance improvements of the system as a whole.

In order to achieve a better performance, the centroid extraction algorithm presented in [44] is used. Considering the observation that segmenting the star pixels from the background through threshold consumes most of the resources during centroid extraction, the strategy employed for in the algorithm is to apply the threshold operation for segmentation accelerated in hardware. This is done as the stream of pixels is being transmitted from the sensor. In our practical implementation, the pixels coming from the sensor through its CSI-2 interface are segmented in FPGA hardware. Subsequently, a new stream constituted of only star pixels is sent to the CPU, which then computes the star centroids.

The centroids are determined by continually filtering the incoming segmented star pixels using a first order Infinite Impulse Response (IIR) filter. The filter is described in Equation (15), where $X_{n}=\left[x_{n},y_{n}\right]$ represents the input, with $x_{n}$ and $y_{n}$ being the coordinates of the pixels, and $Y_{n}$ is the output. The gain $G_{n}$ was defined by Equation (16). According to [44], the optimal value of the *a* constant, selected to minimise the positional error, was found to be 0.8.

(15) $$Y_{n}=G_{n}\times X_{n}+\left(1-G_{n}\right)\times Y_{n-1}$$

(16) $$G_{n}=a^{n}$$

Time is saved in development, when compared to a pure FPGA implementation, by targeting only the bottleneck of the centroiding step in hardware. The remaining operations are still performed in software using the C++ language. A pure software implementation of the new algorithm was also made to serve as a ground truth for the comparisons. By exploring the verification platform’s co-verification functionality, it was possible to use the same test bench to ensure that the implemented centroid extraction algorithms were performing correctly. This was done by comparing the pure software with the hybrid version results and the reference algorithm (Region Growing). Small changes are expected due to the reference algorithm’s different nature and due to different numeric precision between software and hardware implementations. The achieved results can be seen in Table 4 and Table 5, where the first three rows are the total number of stars, the identified stars (true and false positives) and the number of true positives respectively. The mean error is the distance, in pixels, between the estimated and the real centroid coordinates. The last row is the runtime of each approach, and confirms that a better execution time was achieved for the hybrid implementation. The tests were performed with 1000 random attitude configurations and two FOVs: $8^{\circ }$ (Table 4) and $15^{\circ }$ (Table 5).

#### 5.2.3. Improving Star Identification Step Performance

One of the big criticisms of the *Grid Algorithm* is that, in its binary form, finding the closest match in the database requires a search which considers all the entries, resulting in O(n) complexity [45]. The effect of this could be seen in Table 3, where increasing the FOV for the same sensitivity settings increases the number of stars in the images, quickly degrading the performance. A strategy to improve the algorithm’s runtime was employed, inspired by the *Geometric Voting Algorithm* [14]. The angle between the star being identified and its closest neighbor (γ) is added as an additional feature, and this feature orders the database of stars. Catalogue search algorithm complexity is then improved from O(n) to O(k), with *k* being the number of possible stars that have a neighbor with inter-star angle within the tolerance for measurement error *e*. The area search is effectively of stars within [γ−e,γ+e]. A binary descriptor is then used to compare the reference star with the candidates within this search area to find the best match.

As could be expected, this modification produced a significant speed up, particularly in cases when lots of stars are present on the scene being processed. Also, for many cases, this modification increased the correct identification rate of the algorithm. This can be explained by the fact that the *Grid Algorithm* depends on the closest neighbour for achieving rotation invariance. Thus, the correct matching of the closest neighbour is a requirement for generating a correct pattern in rotation. Restricting the search for patterns that have the closest neighbour star within the acceptable angular error range excludes patterns that are very unlikely to be correct, thus increasing the likelihood of correct identification. Table 6 and Table 7 show some comparison data for 8 and 15 degrees of field of view (FOV), respectively, comparing the modified algorithm with different error ranges being considered. The first three rows are the total number of stars of the test, the identified stars (true and false positives) and the true positives, respectively. Speed ups as high as 9.5 times were observed. Thanks to the proposed algorithm’s scalability, even higher speed ups could be achieved as the mean number of stars increases.

The acceptable error *e* parameter should be selected with a high enough value in order to allow 2D position changes of the projections of the stars in the image sensor. Variations on position can be expected due to noise, as described previously in Section 4.4. On the other hand, choosing higher values of *e* have a significant impact in performance, as it could be seen in Table 6 and Table 7. This happens because the database area [γ−e,γ+e] that is being considered is larger, and the search is still being done linearly inside of it. Therefore, the sweet spot of the acceptable error *e* parameter should be high enough in order to allow the presence of positional errors, but low enough to make its introduction useful for enhancing runtime speeds.

**Figure 6:**
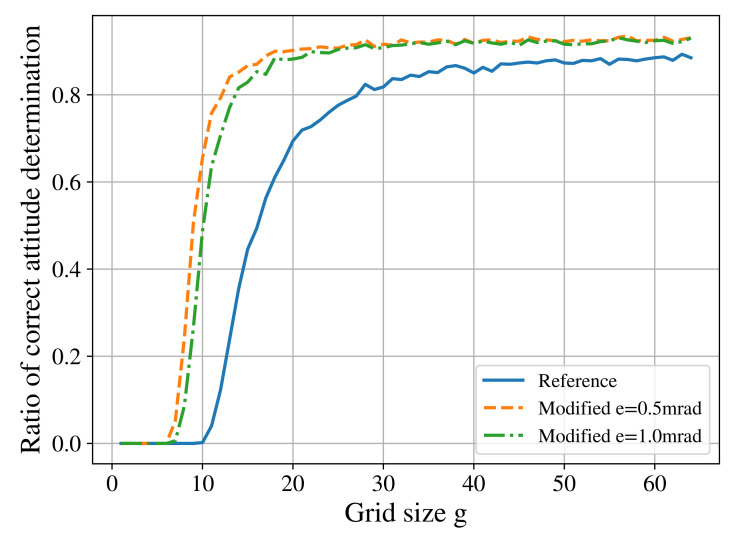
Ratio of correct attitude quaternion determination for different grid sizes. Here, a field of view of 8° is considered.

Considering the complete software stack of the star tracker, for the cases when the addition of the closest neighbour angle (γ) feature increased the number of correctly identified stars, a higher number of correct attitude quaternions was also achieved. An important consequence for hardware requirements is that good identification rates can be realized with a smaller database. As the database size grows proportional to the square of the grid size *g*, the space freed in the database can be easily greater than the space required for the newly added feature. Figure 6 demonstrates this change.

Since the modified version of the *Grid Algorithm* being evaluated could be run in less time and showed better identification rates, it is easy to jump to the conclusion that it is an improvement over the original version. However, as can be seen later in Section 5.3, it requires some precautions to ensure that this stands true in real-world conditions.

#### 5.2.4. Improving the Scoring Function

The score used for the Grid algorithm does not consider false positives, false negatives or true negatives, and is based only on true positive values, as it is based on the binary AND operator. False negatives are difficult to be measured in the system, as non-detections can mistake out-of-image areas (since rotation and translation operations are applied to the image).

Therefore, we investigated the effects of including the false positive information for the scoring process of the Grid algorithm’s binary implementation. The number of false positives can be quickly and reliably obtained by comparing the Population Count (Popcount function, which counts the numbers of ones in a binary word) in the binary descriptor of the sensor image with the Population Count applied to the result of the AND operation between the sensor binary descriptor and the catalogue descriptors. This can be seen in Equation (17). The second term of the operation is already known in the standard Grid algorithm. In the equation, S correspond to the sensor descriptor, D correspond to the current database descriptor being scored, and the AND operation is done bitwise.

(17) Falsepositives=Popcount(S)−Popcount(S∧D).

Or, in other words, if we subtract the number of common stars in both descriptors from the total number of stars detected by the sensor, the result corresponds to the false positives. By decreasing the scoring proportionally to the number of false positives, there was an increase of 1.5% on the number of correctly identified attitude estimates of the Grid algorithm’s binary implementation. As the extra operation are done in the most critical loop of the star identification step, the impact on performance is significant. The runtime increased around 50% as a tradeoff when achieving this better attitude determination rate.

### 5.3. Launch Environment Tests and Focal Length Noise

**Figure 7:**
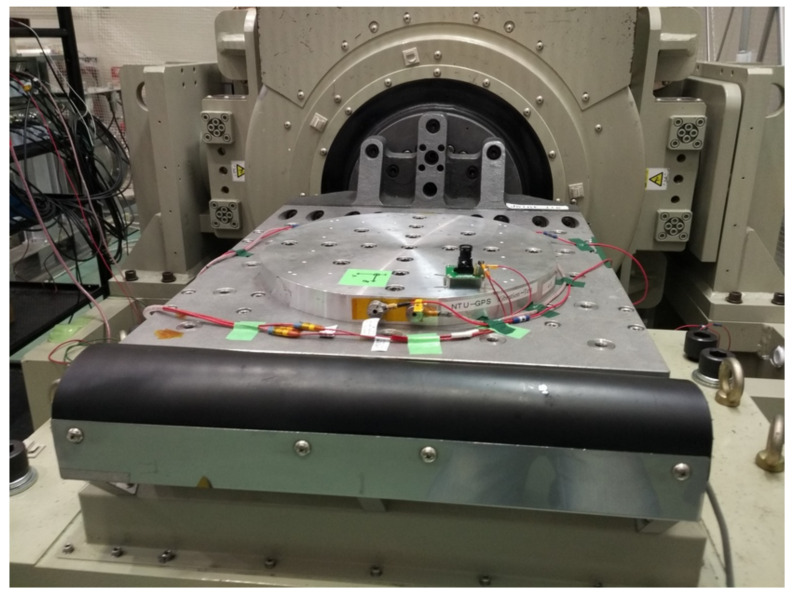
Camera module undergoing vibration tests.

This subsection explains how the UVM-SystemC verification platform can be combined with data of the hardware’s environment tests. This connection with a real application can is used for illustrating purposes. An optical system composed of the image sensor, board and lens, attached to the board using an M-12 lens mount (mechanical interface between the lens and the camera body) was subject to mechanical tests to simulate the environmental conditions of a small satellite launch. The tests, which the optical system was submitted, were the quasi-static load test, random vibration test, and shock test. Before and after each test, a modal survey was also made. All of the tests follow the ISO 19683 recommended levels [46], and were performed in the three coordinates (*x*, *y* and *z*). The DUT can be seen undergoing the vibration tests in Figure 7.

Three functional tests were performed in order to ensure that the system was working properly. One was performed before the vibration tests (quasi-static load and random vibration), the second between the vibration and shock tests, and the last after the shock tests. For the procedure, the optical system captured a chessboard pattern from multiple angles, and the images were used to perform camera calibration of the optics using Zhang’s algorithm [47], through OpenCV’s implementation. Thus, it was possible to measure how the lens’s focal length could change in spacecraft launch conditions. The results can be seen in Table 8.

**Figure 8:**
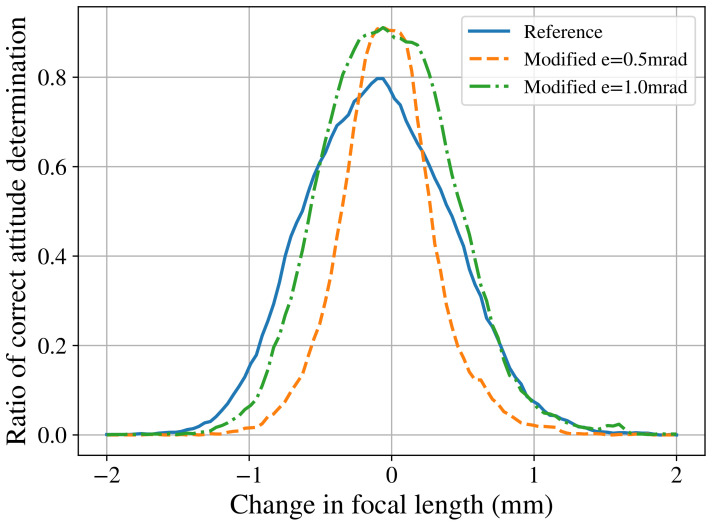
Effect of focal length deviation on the performance of reference and modified algorithms. Here, a field of view of 8° is considered.

Using the verification platform, the focal length was changed from the initial value within the range [−2,+2] mm, and the percentage of correct attitude quaternions was obtained, which is shown in Figure 8.

As can be seen in the Figure 8, a direct consequence of selecting a lower value of the acceptable error *e* is a smaller tolerance to 2D positional noise. This happens when the added noise changes the 2D position of the nearest neighbors are enough to make them fall outside of the area being searched in the database, thus preventing correct matches. The difference in the peak values at zero noise is a consequence of what was previously observed in Table 6. Since the number of correctly matched stars increases when the *e* parameter is used to reduce the database search area, the rate of correctly determined attitude quaternions also increases.

Even though the number of samples of Table 8 is not enough to predict the system’s focal length variation during launch conditions, it gives a general idea of what can be expected. By analysing Figure 8, it can be concluded that it is necessary to take precautions to improve the mechanical construction of the star tracker being designed and/or to recalibrate the system after launch. It was found that the screws present in the M-12 lens holder allow for the lens to change its focal length significantly. Also, in some cases, depending on the parameters chosen, the modified star identification algorithm’s loss of performance can be worse than the reference algorithm as the difference in focus increases. If that is the case, the mentioned precautions become even more necessary.

**Figure 9:**
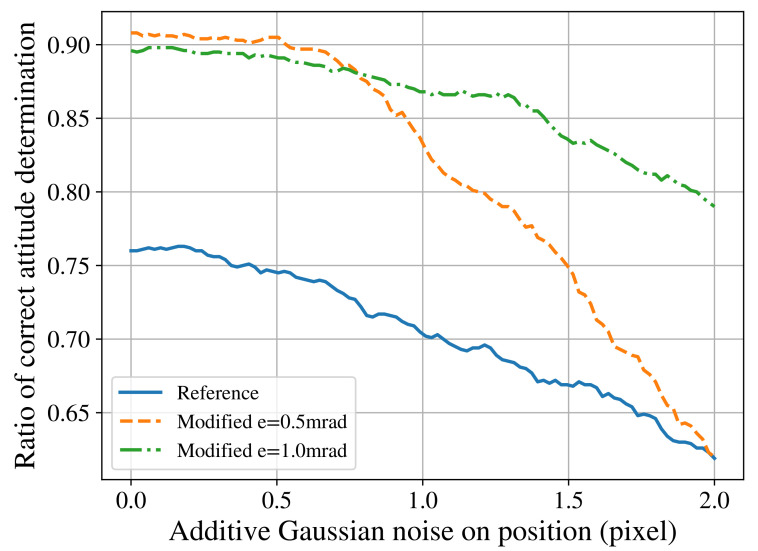
Effect of noise on the 2D position of stars on the performance of reference and modified algorithms. Here, a field of view of 8° is considered.

As other types of noises could also cause a similar behaviour when lower values of *e* are used, a generic 2D positional noise was also analysed. Figure 9 was generated by adding direct random 2D noise modelled by zero mean addictive Gaussian noise. It shows that, in fact, the system has increased sensibility to all noises that might change the position of the projected stars on the sensor for lower values of e=0.5, but this can be mitigated when selecting a more conservative value of e=1.0.

This exemplifies how the verification platform can be used to simulate the influence of specific noise on a given system. This information can then be used to review the design requirements and refine future instances of it. Different star tracker systems might have very different optical parameters, which would require specific tailored simulations for specific noise injection. With the flexibility of the platform’s black-box structure, its universal interfaces, and the ability to adapt its source code to different requirements, it is possible to reuse it to simulate a variety of different noises. These noises can be applied to different systems with heterogeneous characteristics.

## 6. Conclusions

This paper proposed a platform of a well-defined black-box structure for star trackers verification, following the Universal Verification Methodology, of which specific knowledge is transferable between different systems and scopes; the modularity of this structure, with incentives the reusability of verification components; the ability to verify star tracker algorithms and their subcomponents separated or acting together; the ability to speed-up and assist the design of software and hardware components throughout a top-down approach supporting hardware-in-the-loop configurations; and the ability to easily reproduce miscellaneous test conditions used in previous researches. These advantages can directly reduce development time and improve the range of effectiveness of the verification procedures.

The verification platform and simulator were used for straightforward tests such as determining the runtime and the number of correctly identified centroids, stars and attitude quaternions. It was also used to controllably inject noise in the system in specialised ways, such as demonstrated in the lens focal length tests. With the platform’s aid, optimisations in software and hardware of the star tracker were achieved, demonstrating that the system’s energy requirements can be potentially reduced. Approximately two orders of magnitude reduced runtime for the centroiding algorithm through partial hardware acceleration in FPGA. The star identification’s runtime was also reduced around one order of magnitude by employing a different catalog lookup strategy.

The star simulator and verification platform’s sharing as free software in a public repository (https://github.com/schulz89/Verification-Platform-for-Star-Trackers) opens up opportunities for future work in the standardization of test procedures. With a more thorough study of which test conditions and procedures could be considered as ideal to perform fair comparisons, and the current degree of automation of the platform, it would be possible to define a standard batch of tests. If the same standard procedures and common verification tools were used in researches, their results could be directly compared. As the number of algorithms increases over time, having many implementations makes the comparison task very difficult, hindering the scientific process. On the other hand, it is simpler to generate compatible data, facilitating all future comparisons. The data used throughout this article were also made available in the public repository, to assist with the application and the learning curve the proposed methodology.

For future works, it is suggested to investigate techniques in order to reduce the gap between design and its verification as the concepts of system design related to star trackers. Also, thermal effects should be considered along with the vibration effects to better cope with the effective operative scenario, along with the optimal trade-off between the number of processed stars and processing time.

## Figures and Tables

**Table 1:** Instances of star simulators in software.

References	Language	PSF	Noise Model	Attitude Repr.
[25]	C	Gaussian	Gaussian	Euler Matrices
[26]	MATLAB	Gaussian	Not described	Quaternion
[13]	MATLAB	Gaussian	Multiple	Quaternion
[27]	C	Gaussian	Gaussian	Euler Matrices
[28]	MATLAB	Gaussian	None	Quaternions
[12]	C++	Gaussian	Gaussian	Euler Matrices
[11]	MATLAB	Gaussian	Gaussian	Euler Matrices

**Table 2:** Entries from Hipparcos and Hipparcos-2 which are of interest, adapted from [32,34].

Symbol	Catalogue	Label	Description	Unit
-	Hipparcos	HIP	Identifier (HIP number)	-
-	Hipparcos	Vmag	Magnitude in Johnson V	mag
-	Hipparcos-2	HIP	Hipparcos identifier	-
α	Hipparcos-2	RArad	Right Ascension, ICRS, 1991.25	rad
δ	Hipparcos-2	DErad	Declination, ICRS, 1991.25	rad
$\mu_{\alpha }*$	Hipparcos-2	pmRA	Proper motion in Right Ascension	mas/year
$\mu_{\delta }$	Hipparcos-2	pmDE	Proper motion in Declination	mas/year

**Table 3:** Runtime test results (Zynq-7000 ARM Cortex A9MP (ARM v71) SoC @ 667 MHz, single thread).

		Runtime [ms]			
	FOV	Centroid	Star ID	Attitude	Total
Average	8°	13.220	1.759	0.052	15.030
Std. Dev.		0.274	1.653	0.019	1.770
Average	15°	13.780	42.710	0.082	56.570
Std. Dev.		0.352	30.500	0.018	30.750

**Table 4:** Comparison of centroid algorithms, with $FOV=8^{\circ }$.

	Region Growing	Proposed (SW)	Proposed (SW + HW)
Total	10,014	10,014	10,014
Identified	9938	9923	9923
Correct	9938	9918	9918
Mean Error [px.]	0.710	0.741	0.742
Runtime [ms]	13.22	13.84	0.107

**Table 5:** Comparison of centroid algorithms, with $FOV=15^{\circ }$.

	Region Growing	Proposed (SW)	Proposed (SW + HW)
Total	35,006	35,006	35,006
Identified	34,529	34,332	34,332
Correct	34,529	34,309	34,309
Mean Error [px.]	0.714	0.738	0.738
Runtime [ms]	13.78	14.64	0.870

**Table 6:** Comparison of star identification algorithms, $FOV=8^{\circ }$.

	Reference	Binary	Binary
		e = 0.5 Mrad	e = 1.0 Mrad
Total	10014	10014	10014
Identified	6545 (65%)	7100 (71%)	7066 (71%)
Correct	6274 (63%)	7023 (70%)	6976 (70%)
Time μ [ms]	1.759 (1.0×)	1.014 (1.7×)	1.716 (1.0×)
Time σ [ms]	1.653	0.653	1.108

**Table 7:** Comparison of star identification algorithms, $FOV=15^{\circ }$.

	Reference	Binary	Binary
		e = 0.5 Mrad	e = 1.0 Mrad
Total	35,006	35,006	35,006
Identified	25,624 (73%)	24,557 (70%)	27,280 (78%)
Correct	25,099 (72%)	24,032 (69%)	26,892 (77%)
Time μ [ms]	42.71 (1.0×)	4.453 (9.5×)	6.965 (6.1×)
Time σ [ms]	30.50	2.414	3.641

**Table 8:** Focal length variation due to launch environment dynamics.

	*f* [mm]	Δf [mm]
Before tests	25.287	0
After vibration	23.968	−1.3190
After shock	24.283	+0.3150

## Abbreviations

The following abbreviations are used in this manuscript:

UVM	Universal Verification Methodology
FPGA	Field Programmable Gate Array
SoC	System-on-Chip
FOV	field of view
DUT	Device under test
VHDL	Very High Speed Integrated Circuit Hardware Description Language
COTS	Commercial off-the-shelf
IIR	infinite impulse response
HDL	hardware description language
CCD	charged coupled device
CMOS	complementary metal–oxide–semiconductor
